# Editorial: Microbial proteases: Biochemical studies, immobilization and biotechnological application

**DOI:** 10.3389/fmicb.2023.1126989

**Published:** 2023-01-27

**Authors:** Benevides Costa Pessela, Débora Noma Okamoto, Hamilton Cabral

**Affiliations:** ^1^Department of Food Biotechnology and Microbiology, Instituto de Investigación en Ciencias de los Alimentos (CIAL), Consejo Superior de Investigaciones Científicas (CSIC), Cantoblanco, Madrid, Spain; ^2^Laboratório Multidisciplinar em Saúde e Meio Ambiente, Departamento de Ciências Farmacêuticas, Universidade Federal de São Paulo, Diadema, Brazil; ^3^Laboratório de Tecnologia Enzimática, Departamento de Ciências Farmacêuticas, Faculdade de Ciências Farmacêuticas de Ribeirão Preto, Universidade de São Paulo, São Paulo, Brazil

**Keywords:** proteases and industrial processes, pathological processes, protease immobilization, microorganism proteases, proteases biochemical studies

Proteases or peptidases are enzymes that hydrolyze the peptide bonds in peptides and proteins. They are widely distributed in all living organisms, accounting for up to 2% of the human genome. Proteases are currently classified into seven families namely, aspartic, cysteine, glutamic, metallo-, asparagine, serine, and threonine peptidases, in addition to those with unknown or mixed catalytic mechanisms. Proteases are involved in several cellular processes, such as in zymogen activation, turnover, and cellular immunity. Microbial proteases have several applications in different industries, thus making up a very important subclass of hydrolases.

Six articles on microbial proteases impacting medicine, food, and biotechnology were published in this Research Topic. Another aspect, also analyzed in these articles, was the immobilization of these proteases to check if it improved their kinetic properties. Their application in various industrial catalytic processes and their influence in general on human life have also been discussed.

Shu et al. developed a novel and efficient mutagenesis protocol for microbial breeding using an atmospheric and room temperature plasma (ARTP) system. ARTP was used to treat spores of strain 3.042 for the selection of high-acid protease producers. With an irradiation time of 150 s at a lethal rate of 90%, 19 mutants with higher acid protease activity were initially selected based on different mutant colony morphology and the ratio of the clarification halo of protease activity to the colony diameter. In this study, acid protease activity measurements revealed that some mutants, such as strain B-2, are characterized by hereditary stability with increased acid protease, neutral protease, and total protease activities of 54.7, 17.3, and 8.5%, respectively, and decreased alkaline protease activity of 8.1%. In summary, the identified mutant strain B-2 exhibits great potential for enhancing insufficient acid protease activity during the middle and later stages of soy sauce fermentation.

Chen H. et al. cloned and expressed a gene encoding alkaline protease (AprBcp) from *Bacillus circulans* R1. The overexpression of this protease was attempted, reaching a maximum activity of 165,870 U/mL after 60 h of cultivation in a batch fed into a 50 L bioreactor. AprBcp was purified, and the biochemical parameters of optimal pH and temperature were determined to be 10 and 60°C, respectively. This enzyme was stable in the range of pH 8.0 to 11.0 and at temperatures between 30 and 45°C. Moreover, Ca^2+^, Mn^2+^, and Mg^2+^ ions improved protease stability. The protease AprBcp has a potential application in the recovery of soy dreg protein, thus providing the possibility of recovering by-products of the food industry and agriculture.

Wasunan et al. used the strain *Bacillus velezensis* PM 35 from an earthworm carcass to isolate proteases used in the digestion of earthworms. These have the potential to act as bioprophylactic agents against liver cancer and oxidative stress, along with activating immune cells. The response surface method was used to optimize the hydrolysis parameters, determined to be a 3% (v/v) E/S concentration ratio and 3 h of hydrolysis time, achieving a total of 24.62% of the protein hydrolysate and a degree of hydrolysis of 85.45%. Peptides with low molecular weights (MW < 3 kDa) obtained by protease action prevented the proliferation of liver cancer cells HepG2 by causing cell death induced by apoptosis. These peptides also exhibited antioxidant properties.

Chen C. et al. cloned, expressed, characterized, and immobilized an esterase to be used in the degradation of pesticides. Esterases have a catalytic triad similar to that of serine proteases, i.e., Ser-His-Asp, and have various applications. Biochemical parameters indicated that Est804 is an alkaline esterase with an optimum pH of 8.0 and optimal temperature estimated at 45°C, and is capable of preserving 80% of its catalytic activity when exposed to 45°C for 16 h. The kinetic parameters of Est804 were Km = 0.613 mM, kcat = 12.371 s-1, and kcat/Km = 20.181 mM-1s-1. The action of esterase on pesticides revealed cypermethrin (CYP), fenpropathrin (FE), and lambda-cyhalothrin (LCT) degradation rates of 77.35, 84.73, and 74.16%, respectively in 30 min.

Ding et al. cloned and expressed a novel esterase, E53, isolated from *Erythrobacter longus*. The authors revealed the structure of E53 and three other variants using X-ray diffraction. Mutagenesis analysis revealed that mutations in the R1 region can regulate the catalytic reaction both in the positive and negative sense. The mutation in the R2 region improved enzymatic activity, and that in the R3 region was associated with the determination of the E53 pH standard. The exchange of N166A in the R3 region revealed a reduction under alkaline conditions. Structural analysis indicated the role of N166 in stabilizing the loop by forming a hydrogen bond with L193 and G233. The biochemical parameters of E53 in the presence of substrate p-nitrophenyl butyrate were estimated at pH 8.5–9.5 and 40°C, respectively. Based on the structural features, the catalytic pocket was defined as regions R1 (catalytic center), R2 (entrance of the pocket), and R3 (end area of the pocket).

Deng et al. developed a fast, sensitive, precise, and economical method for the detection of *M. pneumoniae* called the dual ERA/CRISPR–Cas12a system. This method showed high specificity and collateral cleavage activity of the LbCas12a protein and, combined with enzyme recombination amplification (ERA) technology, has strong amplification capacity. The results revealed that ERA/CRISPR–Cas12a fluorescence and the dipstick system were able to detect *M. pneumoniae* at titers as low as 1 and 100 copies/mL, respectively. Thus, this study established a portable, fast, low-cost, ultrasensitive, and precise method for the early and rapid diagnosis of *M. pneumoniae*.

In conclusion, all the works published in this Research Topic are important and cover the use of proteases and other enzymes in various domains of life, with a focus in the areas of medicine, food, and biotechnology. In this Research Topic, new enzymes were discovered, and the use of the atmospheric and room temperature plasma system (ARTP) technique was employed to improve the expression process of proteases. Application studies were carried out in the field of biotechnology, such as the use of proteases for application in the recovery of soy-free protein and in protein degradation, obtaining peptides with biological activity. Also, the dual ERA/CRISPR–Cas12a system was developed for the diagnosis and detection of *M. pneumoniae*. Other applications, such as the degradation of pesticides, were carried out by esterase, which has a catalytic triad of serine proteases. Thus, this RT contributed to the field of enzymology by presenting new techniques and applications of enzymes. However, by using transcriptomic techniques, we still need to advance further in other techniques, such as enzymatic engineering and the discovery of new genes that translate other enzymes. This RT opened new perspectives for the field of enzymology and its applications.

## Author contributions

All authors listed have made a substantial, direct, and intellectual contribution to the work and approved it for publication.

